# Using clinical notes to identify children with speech-language delay and understand differences in diagnostic timing

**DOI:** 10.1093/jamiaopen/ooag090

**Published:** 2026-06-12

**Authors:** Jiang Shu, Danai Kasambira Fannin, Geraldine Dawson, Gary Maslow, Matthew M Engelhard, Lauren Franz, Benjamin A Goldstein

**Affiliations:** Department of Biostatistics and Bioinformatics, Duke University, Durham, NC 27705, United States; Department of Communication Sciences and Disorders, North Carolina Central University, Durham, NC 27705, United States; Department of Head and Neck Surgery and Communication Sciences, Duke University School of Medicine, Durham, NC 27705, United States; Department of Psychiatry and Behavioral Sciences, Duke University, Durham, NC 27705, United States; Department of Psychiatry and Behavioral Sciences, Duke University, Durham, NC 27705, United States; Department of Pediatrics, Duke University School of Medicine, Durham, NC 27705, United States; Department of Biostatistics and Bioinformatics, Duke University, Durham, NC 27705, United States; Department of Psychiatry and Behavioral Sciences, Duke University, Durham, NC 27705, United States; Department of Biostatistics and Bioinformatics, Duke University, Durham, NC 27705, United States; Department of Pediatrics, Duke University School of Medicine, Durham, NC 27705, United States

**Keywords:** electronic health records, speech-language delay, Natural Language Processing

## Abstract

**Objective:**

Speech-language delay (SLD) is a developmental condition often identified by pediatricians. While early intervention is recommended, it is common for pediatricians to take a “watch and wait” approach. We sought to assess whether there is discordance between documented concerns and diagnostic coding of SLD and whether discordances differ based on sociodemographic, clinical, and service utilization characteristics.

**Materials and methods:**

We generated an age, sex, payer and race/ethnicity matched cohort of children with and without coded SLD to train a BioClinicalBERT natural learning processing (NLP) model to identify SLD in clinical notes. We applied the model to a population-based test set of well-child visits with no prior SLD diagnosis. We analyzed factors for encounters where SLD was mentioned, but there was no associated ICD-10 code present.

**Results:**

The model attained AUCs of 0.98 (internal validation) and 0.99 (population-based test set). Among encounters where notes documented concerns, only 52% had an associated ICD-10 code. Among the remainder, 39% subsequently received a code (follow-up period up to 650 days), indicating at least 26% of SLD encounters have delayed diagnostic coding. Younger and privately-insured children were less likely to have an ICD-10 code when SLD was documented in notes. Younger children with more outpatient visits were more likely to receive a future SLD diagnosis.

**Discussion:**

NLP can effectively recognize SLD concern in clinical notes, helping to capture those with early documented concerns. Gaps between documentation and coding could suggest “watch and wait” approaches.

**Conclusion:**

Real world SLD research should consider potential discordance between documented concerns and diagnostic codes.

## Background and significance

Speech-language delay, a developmental condition impacting one in five children, can be diagnosed between 18 and 24 months of age when a child does not meet expressive language milestones.^1^ While the vocabulary of children with speech-language delay may significantly improve after 3 years of age without treatment,^2^ difficulties in daily communicative interactions and learning may persist, even for so-called “late-bloomers,” suggesting that for a subset of children with speech-language delay, early identification and intervention remain critical to mitigate long-term impact.^3–5^ Early intervention for speech-language delay is feasible for caregivers to implement, and impacts not only child expressive language, but also cognitive abilities and adaptive behaviors.^6–13^ Importantly, up to 50% of children who present with speech-language delay have co-occurring neurodevelopmental conditions that may benefit from early intervention.^14–19^ As such, the American Academy of Pediatrics recommends prompt referral for speech-language evaluation, audiology, and early intervention when developmental concerns are present, and the 2024 US Preventive Services Task Force Recommendation Statement does not discourage referral when developmental concerns already exist.^20^^,^^21^ However, it is common for pediatricians to take a “watch-and-wait” approach^22^^,^^23^ and qualitative studies indicate that caregivers receive mixed or inconsistent guidance about early language concerns, and experience delays in referral and service initiation.^24–27^

Previous speech-language delay research has largely relied on data from relatively small cohort and intervention studies.^28^ While these data are of high quality, the smaller sample sizes, shorter follow-up times, and higher cost of data accrual limit their utility. Moreover, studies of recruited cohorts may not be representative of broader pediatric populations that include minoritized and underserved communities.^29^ The use of electronic health record (EHR) databases offers a way to address these gaps, and present a unique opportunity to study large, representative cohorts of children, over long periods of time through repeated contacts with the healthcare system, to better understand speech-language delay. This understanding could help connect the right child with the right intervention at the right time. While there have been successful EHR studies of neurodevelopmental conditions, there are very few studying speech-language delay, and those that do rely on International Classification of Diseases (ICD) diagnostic codes to identify the condition.^28–30^ Since diagnostic codes are used to initiate services, it is likely that such codes are coded after speech-language delay concern is recognized by the caregiver or clinician. It is possible that discordances exists between when caregivers first share concerns about their child’s development, and diagnosis of speech-language delay through ICD codes.^31^^,^^32^ Furthermore, factors such as child sex, race, ethnicity, primary home language, and insurance status may affect the diagnosis and management of developmental conditions, including speech-language delay.^28^^,^^33^^,^^34^ This suggests that while structured EHR data can be used to study speech-language delay, there is ample opportunity to leverage and improve its usage.

## Objective

To expand upon ways to identify speech-language delay using EHR data, clinical documentation in provider notes that capture concerns discussed during an office visit can be examined via Natural Language Processing (NLP). NLP methods have previously been used in a variety of EHR-based phenotyping studies,^35–39^ and more specifically, have successfully been applied in the study of neurological and neurodevelopmental conditions, including epilepsy and autism.^35^^,^^40–42^ The goal of this study is to use NLP to evaluate clinical documentation in provider notes to identify children with speech-language delay concern. Further, we explore whether there is a discordance between documented concern of speech-language delay and ICD based diagnosis, and whether these discordances differ based on characteristics. We hypothesize that there will be a subset of children for whom concerns of speech-language delay are documented in clinical notes but not captured through ICD diagnostic coding.

## Materials and methods

### Data source

In this observational, retrospective study, we used EHR data from the Duke University Health System (DUHS), stored in our clinical research data warehouse.^43^ DUHS consists of three hospitals and over 150 clinics that have used an integrated Epic^44^ EHR platform since 2013. Approximately 85% of children in Durham County, which is a racially and socioeconomically diverse county, receive primary care services through DUHS.^45^

### Cohort selection and data abstraction

We constructed two, non-overlapping, analytic cohorts. We first constructed a learning cohort of children with speech-language delay matched to controls. Eligible cases had to: (1) be born at DUHS after January 1, 2016; (2) have at least two encounters with recorded ICD-10 codes for speech-language delay (Table S1); (3) have the first diagnosis occurring during a well-child visit between 12 months of age and 36 months of age and prior to December 31, 2022; (4) have a clinical note at the diagnostic well-child visit. For each case encounter, we identified a control well-child visit from a child that never received a speech-language delay diagnosis matched by sex, payer type, race/ethnicity and age.

Next, we generated a population-based test set. This test set included all well-child visits in 2023 where: (1) the child had no prior speech-language delay diagnosis, (2) it was the child’s first well-child visit in the year, (3) the well-child visit occurred between 12 months and 36 months, (4) the well-child visit had a clinical note.

Finally, we extracted the unstructured note text, along with key structured factors. We used clinical notes associated with pediatric well-child visits (with same templates for all well-child visits), which include sections for history of present illness, physical exam, and assessment/plan. A data entry thus contains note text, sociodemographic (age, sex, race/ethnicity, spoken language, payer type, area deprivation index), clinical (history of motor delay, developmental delay, or autism spectrum disorder) and service utilization (number of previous well-child visits, number of outpatient visits in previous 12 months) information.

### Classifying late talking encounters

We used an 80/20 split to divide the learning data into training and test sets. We abstracted the clinical notes from each case-control pair. We used BioClinical-BERT (BCB)^46^—a transformer-based language model pre-trained on clinical text—to convert encounter notes into dense vector representations (embeddings). BCB is pre-trained on a large corpus of both biomedical literature and clinical notes, making it a well-suited hybrid for our task of analyzing clinical documentation. While more recent large language models exist, BCB provides a powerful and established architecture that balances high performance on clinical tasks with the computational feasibility required for fine-tuning and subsequent resource-intensive interpretability analyses. These embeddings were then input into a classification layer; we fine-tuned the entire BCB model to predict the probability that an encounter’s clinical note reflected speech-language delay issues. A decision rule was applied to these probabilities to classify each encounter (ie, late talking-classified or late talking- not classified). Notes exceeding the model’s maximum input length were split into overlapping segments (chunks) with overlapping windows. We applied a multiple instance learning (MIL) framework, where an encounter was labeled positive if any chunk contained relevant information.^47^ All chunks from each encounter pair were processed together in the same batch and a max pooling operation across all logits was used within each encounter and the maximum logit was then used to compute the loss and update model parameters. We used five-fold cross-validation on the training set (80%) to find the best-performing fine-tuned BCB model, The final hyperparameter setting, determined through cross-validation, included three training epochs, learning rate of 1e−05, dropout rate of 0.1, chunk size of 512 tokens, and an overlap of 128 tokens. All hyperparameter options evaluated during model tuning are detailed in Table S2. We assessed the model performance in the held-out test set (20%).

### SHapley additive exPlanations (SHAP) phrase-level importance analysis

To identify meaningful multi-word expressions indicative of speech-language delay, we used SHAP 0.51.0,^48^ a unified framework that attributes a model’s prediction to its input features. For transformer-based text classifiers, the SHAP library applies PartitionExplainer, which computes Owen values^49^ over a hierarchical partition tree rather than marginal Shapley values over independent tokens. The partition tree groups positionally adjacent tokens into nested coalitions, and the explainer masks tokens in coalition-coherent groups during attribution. This addresses a key limitation of standard SHAP for contextual representations—that the assumption of feature independence does not hold at the token level—by replacing it with a coalition-aware masking scheme in which tightly co-occurring tokens are masked together rather than in isolation. To surface interpretable multi-token expressions, we extracted phrase-level attributions directly from the tree’s internal nodes—each corresponding to a variable-length contiguous token coalition that the explainer evaluated as a unit.

### Population based assessment

We next applied the learned classification model to the population-based classification task. Using just the clinical notes, we classified any encounter as speech-language delay whenever the model-predicted probability of speech-language delay was greater than 90%. This high threshold was selected to prioritize precision, ensuring that encounters classified as positive had strong textual evidence of speech-language delay, thereby minimizing false positives in our analysis of assessing discordance. Then we determined whether the encounter had an ICD-10 code for speech-language delay. We classified each encounter into one of four categories based on the presence or absence of both model-detected speech-language delay concern and ICD-10 codes for speech-language delay, then compared differences across these groups. To assess sociodemographic, clinical, and service utilization differences between children in each of these four categories, we calculated *P*-value based on chi-square tests for categorical factors and ANOVA for continuous factors. To assess differences in diagnostic coding among encounters identified as speech-language delay based on clinical documentation, we regressed the presence of a speech-language delay-related ICD-10 code onto the sociodemographic, clinical, and service utilization factors. We estimated odds ratios (ORs) and 95% confidence intervals (CIs) for: (1) marginally unadjusted; (2) each predictor adjusted for sex and age; and (3) fully adjusted estimates, where all covariates were included in a single multivariable model. Finally, we assessed whether a discordance between clinical documentation and use of an ICD-10 code was indicative of a delay in coding. Among encounters that lacked an ICD-10 code yet were classified by our model as speech-language delay, we extracted data through December 31, 2024, to determine whether the child received a future speech-language delay diagnosis. We used Cox proportional hazards regression, a statistical method for analyzing time-to-event data, to assess factors associated with the time to a speech-language delay diagnosis. This model is appropriate for assessing which patient characteristics are associated with a sooner or later diagnosis, accounting for the fact that not everyone will receive a diagnosis, that is, be censored. The survival time was calculated starting from the date of the initial encounter where speech-language delay was identified by the NLP model but not coded by a speech-language delay ICD-10 code. Hazard ratios (HRs) and 95% CIs were estimated using the same three approaches as above.

NLP analyses were conducted in Python 3.11.9^50^ and statistical analyses were conducted in R 4.1.3.^51^ The study was approved by Duke’s Institutional Review Board with consent waived due to minimal risk.

## Results

**Figure 1.**
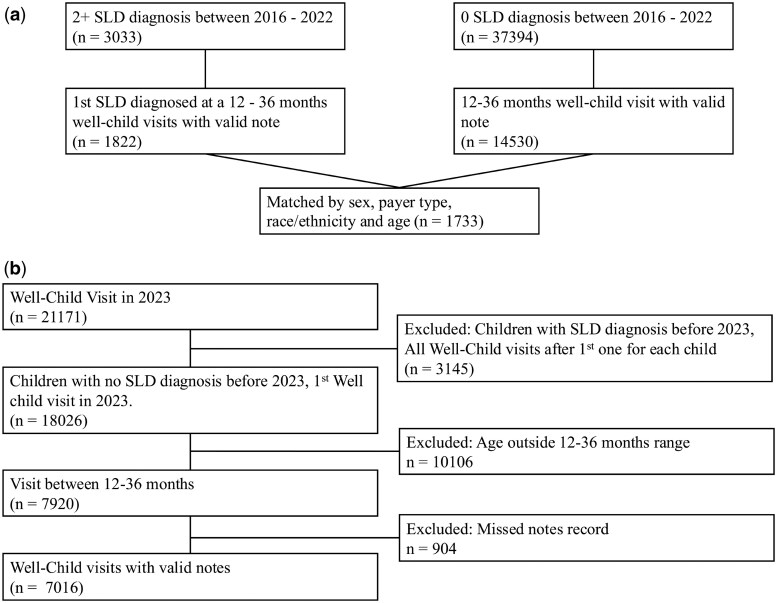
Consort diagram for the creation of (a) the learning set used to develop the NLP classifier and (b) the population test-set. The diagram details inclusion and exclusion criteria, cohort sizes, and key stratification steps.

For the NLP model training, we identified 1822 eligible speech-language delay encounters (Figure 1a). Of these, we were able to find age, sex, payer and race/ethnicity matches for 1733 (95.1%) children. Clinically, children with a speech-language delay diagnosed encounter were more likely to have a history of motor or developmental delay (Table S3).

**Figure 2.**
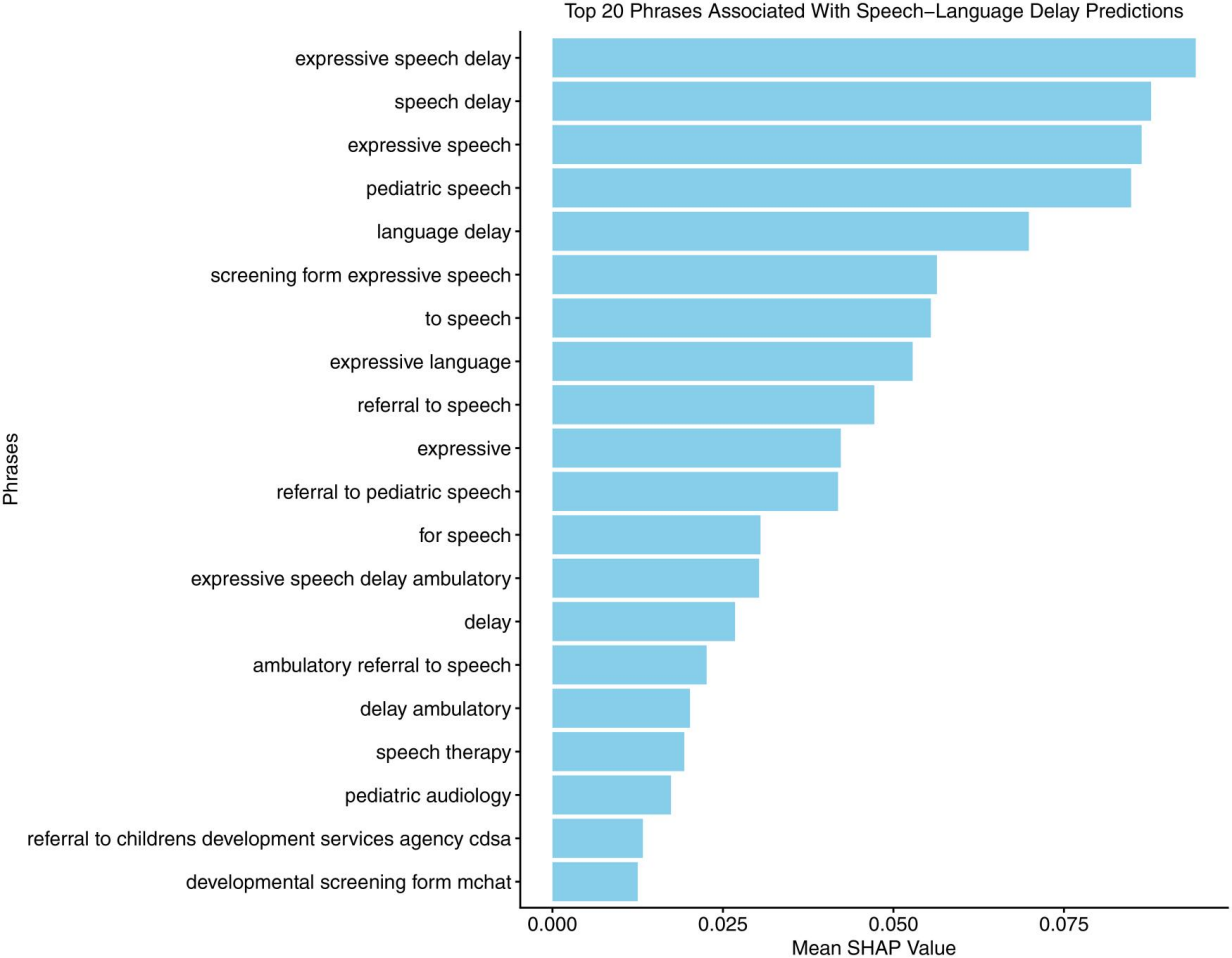
Top 20 phrases associated with speech-language delay, ranked by mean Owen value (SHAP). Phrases correspond to internal nodes of the partition tree produced by PartitionExplainer, representing variable-length contiguous token coalitions evaluated as units by the explainer. Most phrases relate directly to documentation of expressive speech, referral pathways, and developmental screening.

The learned classification model effectively identified speech-language delay encounters based on clinical notes, achieving an AUC of 0.98 on the held-out test set (Figure S1). The top 20 phrases most predictive of speech-language delay classification revealed clinically meaningful language patterns (Figure 2). SHAP values represent a feature’s additive contribution to the model’s predicted probability of speech-language delay. All top-ranked phrases had positive Owen values, indicating they consistently pushed the model’s prediction toward a speech-language delay classification. This reflects the nature of the task, in which the presence of specific clinical language is the primary signal and its absence defines the control group. The aggregate magnitude of these phrase-level contributions is sufficient to drive the model’s high-confidence predictions, confirming that decisions are grounded in clinically relevant reasoning. Phrases such as “expressive speech delay,” “speech delay,” and “expressive speech” had the highest mean Owen values, indicating the strongest associations with model predictions of speech-language delay.

**Figure 3.**
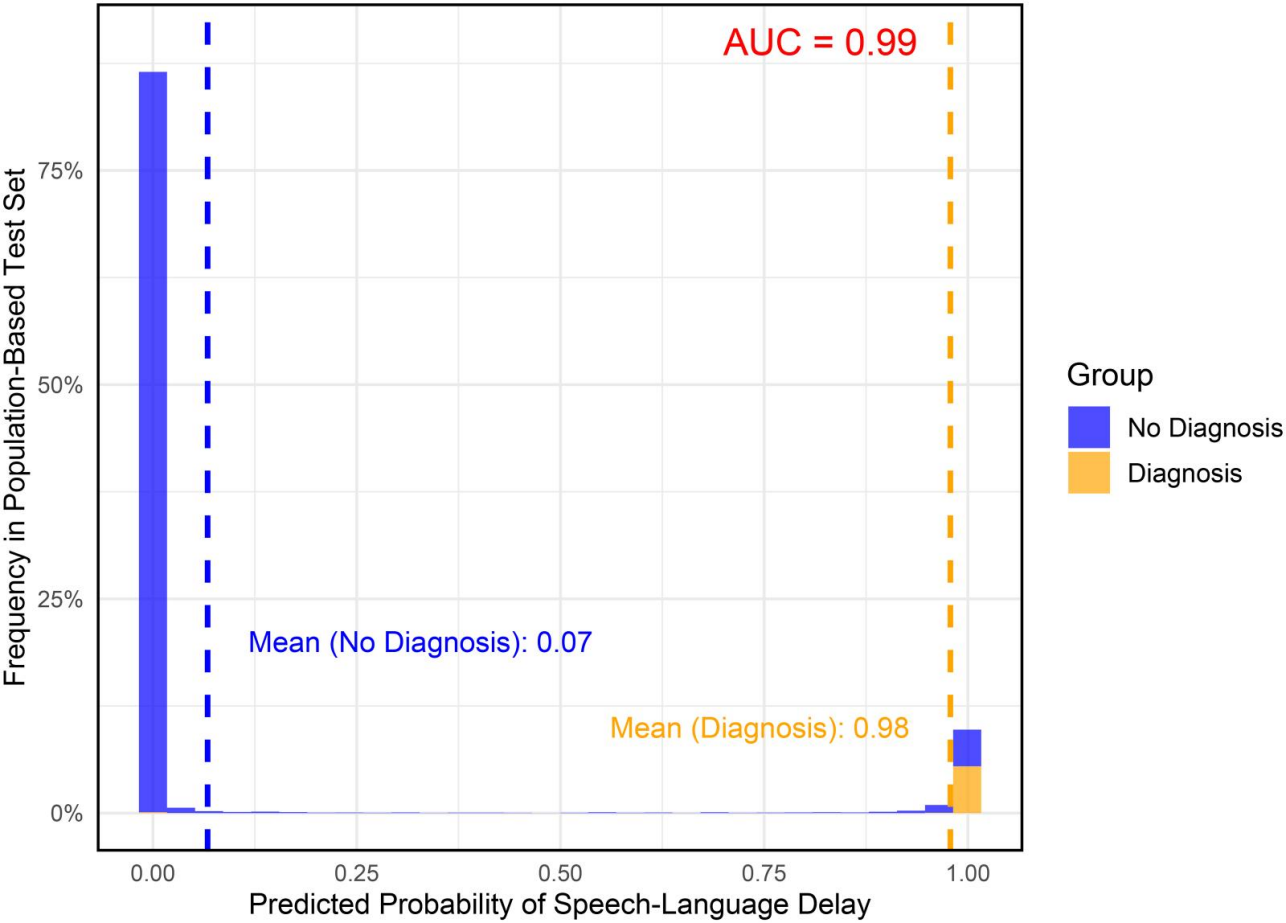
Distribution of predicted probabilities for speech-language delay based on clinical documentation. Blue bars represent where there is no associated ICD-10 code, while yellow bars represent where there is an associated code. Overall, the generated model is very confident (most predicted probabilities are close to 0 or 1) and accurate. However, there is a noted subset of encounters where there is evidence of speech-language delay documentation but no associated code.

For the population-based test set, we identified 7016 eligible Well Child visit encounters (Figure 1b). We applied the model to these encounters, and the model achieved a similarly high concordance with a speech-language delay ICD-10 code (AUC = 0.99). As shown in Figure 3, the model demonstrates strong confidence in its predictions, with the majority of encounters assigned having either very high or very low probabilities.

We classified any encounter with a probability greater than 0.90 as a speech-language delay encounter and stratified the population-based test set based on the classification and receipt of an ICD-10 code (Table 1). Children without speech-language delay are demographically similar to children receiving care at DUHS from Durham County.^45^ Overall, 725 (10.3%) encounters were classified as speech-language delay based on clinical notes and, of those, 380 (52.4%) had an associated speech-language delay ICD-10 diagnosis. Very few encounters (*n* = 9), had an ICD-10 code without related clinical documentation. Among classified encounters, those without an ICD-10 code involved children who were younger, had more total outpatient visits in the previous 12 months, were Non-Hispanic White, on private insurance, had more co-occurring neurodevelopmental conditions, and lower area deprivation index (ADI) scores.

**Table 1.** Characteristics of population-based test set stratified by notes-diagnosis levels.

Variable	Classified + diagnosis	Classified + no diagnosis	Not classified + diagnosis	Not classified + no diagnosis	*P*-val
*N*	380	345	9	6282	
Age in months [Q1, Q3]	24.1 [18.4, 30.6]	18.2 [12.8, 24.9]	26.0 [17.8, 33.4]	18.0 [12.4, 26.7]	<.001
WC visits [median (IQR)]^a^	8.0 [6.0, 10.0]	7.0 [6.0, 8.0]	8.0 [6.0, 10.0]	7.0 [6.0, 10.0]	.001
12-month OP visits [median (IQR)]^b^	5.5 [3.0, 10.0]	8.0 [4.0, 16.0]	3.0 [3.0, 12.0]	8.0 [5.0, 13.0]	<.001
Sex (%)					<.001
Male	231 (60.8)	199 (57.7)	6 (66.7)	3008 (47.9)	
Female	149 (39.2)	146 (42.3)	3 (33.3)	3274 (52.1)	
Race/Ethnicity (%)					<.001
NH-White	86 (22.6)	124 (35.9)	5 (55.6)	2515 (40.0)	
NH-Black	135 (35.5)	101 (29.3)	4 (44.4)	1683 (26.8)	
NH-Asian	15 (3.9)	17 (4.9)	0 (0.0)	368 (5.9)	
Hispanic	96 (25.3)	61 (17.7)	0 (0.0)	1075 (17.1)	
Others	48 (12.6)	42 (12.2)	0 (0.0)	641 (10.2)	
Language (%)					.008
English	328 (86.3)	306 (88.7)	9 (100.0)	5700 (90.7)	
Spanish	50 (13.2)	32 (9.3)	0 (0.0)	532 (8.5)	
Others	2 (0.5)	7 (2.0)	0 (0.0)	50 (0.8)	
Payer (%)					<.001
Private	119 (31.3)	165 (47.8)	2 (22.2)	3578 (57.0)	
Public	255 (67.1)	175 (50.7)	7 (77.8)	2607 (41.5)	
Self-pay or special	6 (1.6)	5 (1.4)	0 (0.0)	97 (1.5)	
Co-occurring conditions					
MD (%)	16 (4.2)	35 (10.1)	0 (0.0)	183 (2.9)	<.001
DD (%)	9 (2.4)	17 (4.9)	0 (0.0)	32 (0.5)	<.001
ASD (%)	0 (0.0)	2 (0.6)	0 (0.0)	1 (0.0)	<.001
ADI (%)^c^					<.001
1	35 (9.2)	42 (12.2)	3 (33.3)	941 (15.0)	
2	69 (18.2)	76 (22.0)	0 (0.0)	1506 (24.0)	
3	45 (11.8)	47 (13.6)	1 (11.1)	814 (13.0)	
4	59 (15.5)	46 (13.3)	1 (11.1)	765 (12.2)	
5	43 (11.3)	35 (10.1)	0 (0.0)	679 (10.8)	
6	23 (6.1)	23 (6.7)	0 (0.0)	267 (4.3)	
7	35 (9.2)	24 (7.0)	1 (11.1)	322 (5.1)	
8	14 (3.7)	13 (3.8)	0 (0.0)	244 (3.9)	
9	26 (6.8)	13 (3.8)	1 (11.1)	287 (4.6)	
10	18 (4.7)	16 (4.6)	0 (0.0)	222 (3.5)	
Unknown	13 (3.4)	10 (2.9)	2 (22.2)	235 (3.7)	

Abbreviations: WC, Well-Child; OP, outpatient; NH, Non-Hispanic; MD, Motor Delay; DD, Developmental Delay; ASD, Autistic Spectrum Disorders; ADI, Area Deprivation Index.

a Number of all previous Well-Child visits.

b Number of outpatient visits in previous 12 months.

c Lower ADI represents wealth or prosperity, while higher ADI reflects deprivation.

Taking the subset of encounters classified as speech-language delay based on clinical documentation, we conducted a multivariable logistic regression analysis, looking at factors associated with receipt of a speech-language delay ICD-10 code. Fully adjusted model results in Table 2 revealed that older children (OR = 1.05, CI, 1.02-1.07) and children on public insurance (OR = 1.58, CI, 1.07-2.33) were more likely to receive a speech-language delay ICD-10 code. Additionally, children with more well-child visits (OR = 1.09, CI, 1.02-1.17), yet fewer overall outpatient visits (OR = 0.98, CI, 0.96-0.99), were also more likely to receive an ICD-10 code.

**Table 2.** Results of Logistic Regression Model for classified encounters.

**Variable** ^a^	**Marginal OR (CI)**	**Adjusted for sex + age OR (CI)**	**Fully-adjusted OR (CI)**
Age in months	**1.07 (1.05-1.09)**	**1.07 (1.05-1.09)**	**1.05 (1.02-1.07)**
WC visits^b^	**1.13 (1.06-1.20)**	**1.05 (0.99-1.12)**	**1.09 (1.02-1.17)**
12-month OP visits^c^	**0.95 (0.94-0.97)**	**0.97 (0.95-0.99)**	**0.98 (0.96-0.99)**
Sex (female)	0.88 (0.65-1.18)	0.88 (0.65-1.20)	0.86 (0.62-1.18)
RACE/ETH (Hispanic)	**2.27 (1.49-3.46)**	**2.12 (1.37-3.27)**	1.68 (0.98-2.90)
RACE/ETH (NH-Asian)	1.27 (0.60-2.68)	1.43 (0.66-3.07)	2.03 (0.88-4.70)
RACE/ETH (NH-Black)	**1.93 (1.32-2.81)**	1.45 (0.98-2.16)	1.10 (0.71-1.73)
RACE/ETH (others)	1.65 (1.00-2.71)	1.40 (0.84-2.35)	1.19 (0.69-2.05)
Language (others)	0.27 (0.05-1.29)	0.30 (0.06-1.53)	0.22 (0.04-1.21)
Language (Spanish)	1.46 (0.91-2.33)	1.46 (0.90-2.38)	0.78 (0.42-1.45)
Payer (public)	**2.02 (1.49-2.74)**	**1.71 (1.25-2.35)**	**1.58 (1.07-2.33)**
Payer (self-pay or special)	1.66 (0.50-5.58)	1.59 (0.45-5.59)	1.45 (0.40-5.29)
MD (yes)	**0.39 (0.21-0.72)**	**0.43 (0.23-0.81)**	0.55 (0.28-1.07)
DD (yes)	0.47 (0.21-1.06)	0.43 (0.18-1.01)	0.51 (0.20-1.29)
ADI^d^: 4-6	1.33 (0.95-1.87)	1.26 (0.89-1.80)	1.21 (0.83-1.77)
ADI^d^: 7-10	**1.56 (1.06-2.29**)	1.41 (0.95-2.11)	1.24 (0.80-1.93)
ADI^d^: Unknown	1.44 (0.61-3.38)	1.47 (0.61-3.55)	1.59 (0.63-4.01)

Bold values indicate statistically significant association. Abbreviations: OR, Odds Ratio; WC, Well-Child; OP, outpatient; ETH, Ethnicity; NH, Non-Hispanic; MD, Motor Delay; DD, Developmental Delay; ADI, Area Deprivation Index.

a Categorical variable reference level: Male for Sex, NH-White for RACE_ETH, English for Language, Private for Payer, No for MD and DD, 1-3 for ADI.

b Number of all previous Well-Child visits.

c Number of outpatient visits in previous 12 months.

d Lower ADI represents wealth or prosperity, while higher ADI reflects deprivation.

**Figure 4.**
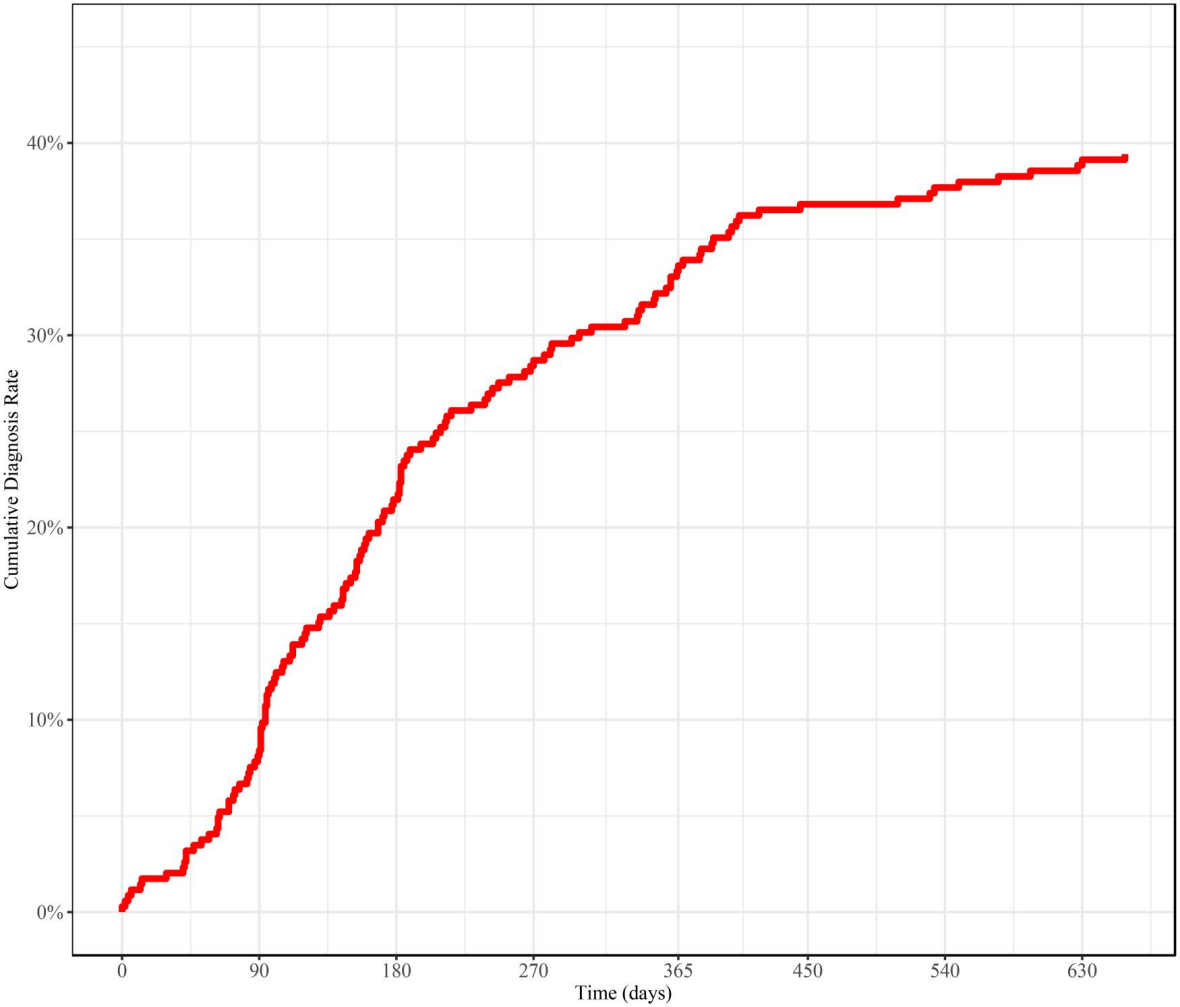
Cumulative incidence plot showing time to formal diagnosis of speech-language delay for the 345 children the model identified as having speech-language delay documentation but no associated ICD-10 code. Clinically meaningful time points (90, 180, 270, 365, etc. days) are marked. The gradual increase in diagnoses over time supports the hypothesis of gap between documented concern and formal coding in this subgroup.

Finally, for the 345 children classified as speech-language delay who did not receive an ICD-10 code, we examined whether they had a diagnosis in a future encounter. Figure 4 presents the cumulative diagnosis rate over time, with 136 (39.4%) children eventually receiving a diagnosis. This suggests that at least 26% of speech-language delay encounters have delayed diagnostic coding. As this is a contemporary cohort, it is noted that, over time, we anticipate that this number will increase as more of these children are diagnosed in the future.

Table 3 presents the results from our Cox regression models assessing factors associated with the time to a future speech-language delay diagnosis for these 345 children. In the fully adjusted model, younger children were somewhat more likely to receive a future diagnosis, although the association was borderline significant (HR: 0.97, 95% CI, 0.95-1.00). This indicates that for each additional month of age, the likelihood of receiving a speech-language delay diagnosis during follow-up decreased slightly. A greater number of outpatient visits in the prior 12 months was also significantly associated with a shorter time to speech-language delay diagnosis (HR: 1.02, 95% CI, 1.01-1.03). While not statistically significant in the fully-adjusted model, it is notable that in marginal and age, sex-adjusted analyses, children with public insurance and those from higher-deprivation neighborhoods (ADI 7-10) received diagnoses more quickly than their privately-insured and lower-deprivation counterparts.

**Table 3.** Cox Proportional Hazards Regression Analysis for potentially-missed children.

**Variable** ^a^	**Marginal HR (CI)**	**Adjusted for sex + age HR (CI)**	**Fully-adjusted HR (CI)**
Age in months	**0.97 (0.95-1.00)**	**0.97 (0.95-1.00)**	**0.97 (0.95-1.00)**
WC visits^b^	**0.93 (0.87-0.99)**	0.95 (0.88-1.03)	0.99 (0.90-1.08)
12-month OP visits^c^	**1.01 (1.01-1.02)**	**1.01 (1.00-1.02)**	**1.02 (1.01-1.03)**
Sex (female)	0.76 (0.54-1.07)	0.77 (0.55-1.09)	0.86 (0.60-1.23)
RACE_ETH (Hispanic)	1.57 (0.97-2.52)	1.57 (0.98-2.52)	1.17 (0.64-2.14)
RACE_ETH (NH-Asian)	1.58 (0.74-3.38)	1.40 (0.65-3.00)	1.44 (0.63-3.29)
RACE_ETH (NH-Black)	1.41 (0.92-2.16)	**1.56 (1.01-2.41)**	1.23 (0.73-2.09)
RACE_ETH (Others)	1.00 (0.54-1.83)	1.06 (0.58-1.94)	1.07 (0.56-2.04)
Language (others)	1.65 (0.52-5.19)	1.42 (0.45-4.50)	1.15 (0.33-4.07)
Language (Spanish)	1.36 (0.79-2.33)	1.39 (0.81-2.38)	1.03 (0.53-2.01)
Payer (public)	**1.70 (1.19-2.43)**	**1.80 (1.26-2.59)**	1.51 (0.95-2.42)
Payer (self-pay or special)	1.40 (0.66-2.97)	1.62 (0.76-3.43)	1.54 (0.69-3.45)
MD (yes)	0.91 (0.51-1.61)	0.89 (0.50-1.57)	0.74 (0.38-1.43)
DD (yes)	1.50 (0.76-2.95)	1.47 (0.75-2.90)	1.00 (0.47-2.16)
ADI^d^: 4-6	1.31 (0.88-1.94)	1.38 (0.93-2.06)	1.26 (0.83-1.92)
ADI^d^: 7-10	**1.79 (1.17-2.74)**	**1.78 (1.16-2.72)**	1.41 (0.87-2.29)
ADI^d^: Unknown	0.27 (0.04-1.92)	0.26 (0.04-1.89)	0.25 (0.03-1.85)

Bold values indicate statistically significant associations. Abbreviations: HR, Hazard Ratio; WC, Well-Child; OP, outpatient; ETH, Ethnicity; NH, Non-Hispanic; MD, Motor Delay; DD, Developmental Delay; ADI, Area Deprivation Index.

a Categorical variable reference level: Male for Sex, NH-White for RACE_ETH, English for Language, Private for Payer, No for MD and DD, 1-3 for ADI.

b Number of all previous Well-Child visits.

c Number of outpatient visits in previous 12 months.

d Lower ADI represents wealth or prosperity, while higher ADI reflects deprivation.

## Discussion

This study leverages EHR data and NLP techniques to identify children with speech-language delay and examine sociodemographic, healthcare utilization, and clinical characteristics associated with the usage of an ICD-10 diagnostic code. We used both a well-matched cohort to train the classifier as well as a more representative population-based cohort to assess its real-world impact. First, we demonstrated that clinical documentation can be used to develop a robust classification model for speech-language delay (AUROC = 0.98). Second, our population-based assessment showed a high degree of discordance between narrative documentation and structured diagnostic coding of speech-language delay, with at least 39% of children whose clinic notes suggest speech-language delay concern later receiving an ICD-based diagnosis.

Real world data sources such as EHRs are important resources for clinical research. However, the fidelity of these sources relies on the consistent documentation of clinical conditions. In general, ICD codes are used to classify diseases and related conditions, provide a standardized and objective framework for documenting diagnoses and support justification for interventions.^52^ However, in pediatric primary care settings, ICD codes are primarily used for reimbursement purposes rather than a tool for clinical documentation. For conditions like speech-language delay, where ICD-code based documentation is often tied to service referral, there may be a difference in time of when concerns are first mentioned and when ICD codes are formally documented. Our results showed that at least 26%, and potentially up to 48%, of children eventually diagnosed with speech-language delay did not receive an ICD-based diagnosis code when concern was first documented, indicative of pediatricians taking a “watch and wait” approach. Others, too, have found that “watch and wait” approaches are common in practice,^22^^,^^23^^,^^53^^,^^54^ even though the evidence assessing early intervention suggest meaningful benefit. However, it is critical to note that a gap between documented concern and formal coding does not necessarily represent a failure to provide care, and future work should examine this explicitly.

We used regression analyses to assess factors related to both a discordance at the initial encounter as well as time to receipt of a formal diagnosis. Our logistic regression analyses found that younger children and children on private insurance were less likely to receive an ICD-based diagnostic code of speech-language delay at a clinical encounter with documented speech and language concerns. These differences may relate to clinician hesitation to diagnose the condition in younger children, as speech and language milestones vary widely at early ages,^55^ in addition to beliefs that children with mild delays may catch up on their own, leading to a watchful waiting approach.^56^ Families with private insurance may also be more likely to seek speech therapy or developmental evaluations outside the healthcare system, leading to fewer formal ICD-10 diagnoses within routine pediatric care.^57^ Additionally, providers may perceive privately insured families as having greater resources to address speech-language delay concerns without a formal diagnosis, leading to diagnostic hesitation compared to publicly insured families, where ICD-coding may be required for service access.^28^ The association between fewer well-child visits and lower likelihood of receiving a code may be explained by reduced opportunities for developmental surveillance and screening that occur during these types of visits.^20^ Fewer well-child visits therefore mean fewer opportunities for developmentally focused clinical observation and discussion of emerging concerns and early detection. Finally, our result that children with a higher number of outpatient visits in the previous 12 months were less likely to receive a code may reflect the presence of ongoing or emergent medical concerns. Clinicians may prioritize these emergent medical issues over developmental surveillance and monitoring during these outpatient encounters.^58^^,^^59^

For children with speech-language delay concerns documented in clinical notes, but no formal ICD diagnosis, Cox-PH model analyses similarly indicated those who were younger, as well as those with a greater number of outpatient visits in the previous 12 months, were more likely to receive a future diagnosis, suggesting a delay in formal coding after first documented concerns. Again, it is not surprising that pediatricians may take a “watch and wait” approach to provide a formal diagnosis even while documenting concerns with younger children. As for children with a higher number of outpatient visits in the previous year, clinicians may eventually assign a diagnostic code once it becomes clearer that persistent speech-language delay are not solely attributable to other conditions.

To mitigate the limitation of relying on ICD-10 codes for identification of children with speech-language delay, our results show that clinical documentation can be used to identify such encounters with high fidelity. A potential circularity of our study is the use of ICD-10 codes to define the positive class for training our NLP model, as opposed to expert-annotated notes. This approach was a deliberate methodological choice, framing the task as a positive-unlabeled learning problem. We designed our case definition—requiring at least two speech-language delay-related ICD codes—to maximize specificity, ensuring our positive examples were highly likely to be true cases. We acknowledge that the control group, defined by the absence of a code, might have low sensitivity and inevitably included some uncoded encounters with concerns documented. However, we believe the model’s high performance on a separate population-based cohort, its ability to identify children who later received a formal ICD diagnosis provide strong evidence that it captured a true clinical signal beyond billing patterns alone. Moreover, the SHAP analysis highlighted phrases whose attributions reflect coherent clinical concepts rather than isolated tokens. These findings provide evidence that the model learns clinically meaningful and interpretable patterns, enhancing trust in its predictions. Many of the top-ranked phrases—such as, “language delay,” and “expressive speech delay,” directly align with terminology clinicians use when documenting language concerns or planning referrals, suggesting that the model’s decisions are grounded in relevant clinical reasoning rather than spurious correlations.

While this study has several strengths, there are some limitations. For one, the study focuses on encounters from a single health system, which may limit the applicability of the results to other geographic regions or settings. In particular, the diagnostic differences may be different in other institutions. Second, it is not clear if the lack of documentation implies a lack of clinical action. Since most referrals to speech services would happen outside of our institution, it is possible such referrals are still occurring, mitigating the negative repercussions of the observed under-coding of late talking. Future work should seek to understand these downstream processes. Finally, it is possible that there are encounters where speech-language delay is being discussed but the provider is not coding it nor are they writing about it in the notes. In such settings, tools like ambient listening may prove useful, as the documentation relies less on an explicit choice by the provider.

## Conclusion

Leveraging data from a large academic health system, we demonstrated that an NLP-based model can effectively detect speech-language delay concerns using clinical notes, further enabling the exploration of discordances between documented concerns and formal coding. This study highlights the usefulness of NLP in analyzing unstructured clinical data, enabling researchers to extract valuable insights that transcend traditional reliance on structured ICD codes. Our findings suggest that younger children and those with more outpatient visits in the previous 12 months are associated with a delay in formal diagnostic coding, perhaps due to “watch and wait” approaches. This highlights a critical limitation of EHR-based research that relies solely on structured codes and demonstrates the value of using NLP to establish another timeline of when concerns are firstly documented. Future work could build upon this by using NLP to extract information on treatment recommendations and referrals to better understand the full clinical management pathway for late talking.

## Supplementary Material

ooag090_Supplementary_Data

## Data Availability

The data underlying this article cannot be shared publicly as they consist of patient electronic health records containing protected health information (PHI). A de-identified dataset may be made available on reasonable request to the corresponding author (Benjamin A. Goldstein, ben.goldstein@duke.edu) and is subject to approval by the Duke University Institutional Review Board (IRB).

## References

[1] Law J , Boyle J, Harris F, et al Prevalence and natural history of primary speech and language delay: findings from a systematic review of the literature. Int J Lang Commun Disord. 2000;35:165-188. 10912250 10.1080/136828200247133

[2] Dale PS , Price TS, Bishop DVM, et al Outcomes of early language delay: I. Predicting persistent and transient language difficulties at 3 and 4 years. J Speech Lang Hear Res. 2003;46:544-560. 14696985 10.1044/1092-4388(2003/044)

[3] Matte-Landry A , Boivin M, Tanguay-Garneau L, et al Children with persistent versus transient early language delay: language, academic, and psychosocial outcomes in elementary school. J Speech Lang Hear Res. 2020;63:3760-3774. 33105083 10.1044/2020_JSLHR-20-00230

[4] Law J , Rush R, Schoon I, et al Modeling developmental language difficulties from school entry into adulthood: literacy, mental health, and employment outcomes. J Speech Lang Hear Res. 2009;52:1401-1416. 19951922 10.1044/1092-4388(2009/08-0142)

[5] Nelson CA , Sullivan E, Engelstad A. Annual research review: early intervention viewed through the lens of developmental neuroscience. J Child Psychol Psychiatry. 2024;65:435-455. 37438865 10.1111/jcpp.13858

[6] Dawson G , Rogers S, Munson J, et al Randomized, controlled trial of an intervention for toddlers with autism: the early start Denver model. Pediatrics. 2010;125:e17–23-e23. 19948568 10.1542/peds.2009-0958 PMC4951085

[7] Hyman SL , Levy SE, Myers SM, COUNCIL ON CHILDREN WITH DISABILITIES, SECTION ON DEVELOPMENTAL AND BEHAVIORAL PEDIATRICS. Identification, evaluation, and management of children with autism spectrum disorder. Pediatrics. 2020;145:e20193447. 31843864 10.1542/peds.2019-3447

[8] Lord C , Rutter M, Goode S, et al Austism diagnostic observation schedule: a standardized observation of communicative and social behavior. J Autism Dev Disord. 1989;19:185-212. 2745388 10.1007/BF02211841

[9] Schreibman L , Dawson G, Stahmer AC, et al Naturalistic developmental behavioral interventions: empirically validated treatments for autism spectrum disorder. J Autism Dev Disord. 2015;45:2411-2428. 25737021 10.1007/s10803-015-2407-8 PMC4513196

[10] Carson L , Baker E, Munro N. A systematic review of interventions for late talkers: intervention approaches, elements, and vocabulary outcomes. Am J Speech Lang Pathol. 2022;31:2861-2874. 36251872 10.1044/2022_AJSLP-21-00168

[11] Wilcox MJ , Kouri TA, Caswell SB. Early language intervention: a comparison of classroom and individual treatment. Am J Speech Lang Pathol. 1991;1:49-62.

[12] Leonard LB , Schwartz RG, Chapman K, et al Early lexical acquisition in children with specific language impairment. J Speech Hear Res. 1982;25:554-564. 7162156 10.1044/jshr.2504.554

[13] Buschmann A , Multhauf B, Hasselhorn M, et al Long-term effects of a parent-based language intervention on language outcomes and working memory for late-talking toddlers. J Early Interv. 2015;37:175-189.

[14] Nitzan T , Koller J, Ilan M, et al The importance of language delays as an early indicator of subsequent ASD diagnosis in public healthcare settings. J Autism Dev Disord. 2023;53:4535-4544. 36222994 10.1007/s10803-022-05757-y

[15] Justice LM , Jiang H, Bates R, et al Language disparities related to maternal education emerge by two years in a low-income sample. Matern Child Health J. 2020;24:1419-1427. 32632843 10.1007/s10995-020-02973-9 PMC7572544

[16] Plug MB , van Wijngaarden V, de Wilde H, et al Clinical characteristics and genetic etiology of children with developmental language disorder. Front Pediatr. 2021;9:651995. 34277514 10.3389/fped.2021.651995 PMC8282268

[17] Hustad KC , Allison K, McFadd E, et al Speech and language development in 2-year-old children with cerebral palsy. Dev Neurorehabil. 2014;17:167-175. 23627373 10.3109/17518423.2012.747009 PMC3732544

[18] Baumer FM , Cardon AL, Porter BE. Language dysfunction in pediatric epilepsy. J Pediatr. 2018;194:13-21. 29241678 10.1016/j.jpeds.2017.10.031 PMC5826845

[19] Solot CB , Sell D, Mayne A, et al Speech-Language disorders in 22q11.2 deletion syndrome: best practices for diagnosis and management. Am J Speech Lang Pathol. 2019;28:984-999. 31330115 10.1044/2019_AJSLP-16-0147 PMC6802924

[20] Lipkin PH , Macias MM, COUNCIL ON CHILDREN WITH DISABILITIES, SECTION ON DEVELOPMENTAL AND BEHAVIORAL PEDIATRICS. Promoting optimal development: Identifying infants and young. Children with developmental disorders through developmental surveillance and screening. Pediatrics. 2020;145:e20193449. 31843861 10.1542/peds.2019-3449

[21] Barry MJ , Nicholson WK, Silverstein M, US Preventive Services Task Force, et al Screening for speech and language delay and disorders in children: US preventive services task force recommendation statement. JAMA. 2024;331:329-334. 38261037 10.1001/jama.2023.26952

[22] Capone Singleton N. Late talkers: why the wait-and-see approach is outdated. Pediatr Clin North Am. 2018;65:13-29. 29173713 10.1016/j.pcl.2017.08.018

[23] Nouraey P , Ayatollahi MA, Moghadas M. Late language emergence. Sultan Qaboos Univ Med J. 2021;21:e182-e190. 34221464 10.18295/squmj.2021.21.02.005 PMC8219342

[24] Ash AC , Christopulos TT, Redmond SM. “Tell me about your child”: a grounded theory study of mothers’ understanding of language disorder. Am J Speech Lang Pathol. 2020;29:819-840. 32348158 10.1044/2020_AJSLP-19-00064 PMC7842869

[25] O’Toole C , Lyons R, Houghton C. A qualitative evidence synthesis of parental experiences and perceptions of parent-child interaction therapy for preschool children with communication difficulties. J Speech Lang Hear Res. 2021;64:3159-3185. 34289311 10.1044/2021_JSLHR-20-00732

[26] Meurer J , Rohloff R, Rein L, et al Improving child development screening: implications for professional practice and patient equity. J Prim Care Community Health. 2022;13:21501319211062676. 34986680 10.1177/21501319211062676 PMC8743928

[27] Nayeb L , Wallby T, Westerlund M, et al Child healthcare nurses believe that bilingual children show slower language development, simplify screening procedures and delay referrals. Acta Paediatr. 2015;104:198-205. 25327143 10.1111/apa.12834

[28] Frelinger C , Gardner RM, Huffman LC, et al Detection of speech-language delay in the primary care setting: an electronic health record investigation. J Dev Behav Pediatr. 2023;44:e196–203-e203. 36978234 10.1097/DBP.0000000000001167 PMC10065357

[29] Goodwin Cartwright BM , Smits PD, Stewart S, et al Time-series analysis of first-time pediatric speech delays from 2018 to 2022. JAMA Pediatr. 2024;178:193-195. 38048098 10.1001/jamapediatrics.2023.5226 PMC10696512

[30] Engelhard MM , Berchuck SI, Garg J, et al Health system utilization before age 1 among children later diagnosed with autism or ADHD. Sci Rep. 2020;10:17677. 33077796 10.1038/s41598-020-74458-2 PMC7572401

[31] Baio J , Wiggins L, Christensen DL, et al Prevalence of autism spectrum disorder among children aged 8 years–autism and developmental disabilities monitoring network, 11 sites, United States, 2014. MMWR Surveill Summ. 2018;67:1-23. 10.15585/mmwr.ss6706a1 PMC5919599 29701730

[32] Zablotsky B , Colpe LJ, Pringle BA, et al Age of parental concern, diagnosis, and service initiation among children with autism spectrum disorder. Am J Intellect Dev Disabil. 2017;122:49-61. 28095057 10.1352/1944-7558-122.1.49 PMC5568529

[33] Constantino JN , Abbacchi AM, Saulnier C, et al Timing of the diagnosis of autism in African American children. Pediatrics. 2020;146:e20193629. 32839243 10.1542/peds.2019-3629 PMC7461218

[34] Gallegos A , Dudovitz R, Biely C, et al Racial disparities in developmental delay diagnosis and services received in early childhood. Acad Pediatr. 2021;21:1230-1238. 34020100 10.1016/j.acap.2021.05.008 PMC9169674

[35] Chen J , Engelhard M, Henao R, et al Enhancing early autism prediction based on electronic records using clinical narratives. J Biomed Inform. 2023;144:104390. 37182592 10.1016/j.jbi.2023.104390 PMC10526711

[36] Spratt SE , Pereira K, Granger BB, DDC Phenotype Group, et al Assessing electronic health record phenotypes against gold-standard diagnostic criteria for diabetes mellitus. J Am Med Inform Assoc. 2017;24:e121–8-e128. 27616701 10.1093/jamia/ocw123 PMC6080723

[37] Chang F , Krishnan J, Hurst JH, et al Comparing natural language processing and structured medical data to develop a computable phenotype for patients hospitalized due to COVID-19: retrospective analysis. JMIR Med Inform. 2023; 11: e46267–e46267. 37621195 10.2196/46267 PMC10466442

[38] Tang M , Goldstein BA, He J, et al Performance of a computable phenotype for pediatric asthma using the problem list. Ann Allergy Asthma Immunol. 2020;125:611-613.e1. 32687988 10.1016/j.anai.2020.07.009 PMC7606382

[39] Hurst JH , Brucker A, Zhao C, et al Use of structured electronic health records data elements for the development of computable phenotypes to identify potential adverse events associated with intravenous immunoglobulin infusion. Drug Saf. 2023;46:309-318. 36826707 10.1007/s40264-023-01276-6

[40] Chafjiri FMA , Reece L, Voke L, et al Natural language processing for identification of refractory status epilepticus in children. Epilepsia. 2023;64:3227-3237. 37804085 10.1111/epi.17789

[41] Decker BM , Turco A, Xu J, et al Development of a natural language processing algorithm to extract seizure types and frequencies from the electronic health record. Seizure. 2022;101:48-51. 35882104 10.1016/j.seizure.2022.07.010 PMC9547963

[42] Zhao M , Havrilla J, Peng J, et al Development of a phenotype ontology for autism spectrum disorder by natural language processing on electronic health records. J Neurodev Disord. 2022;14:32. 35606697 10.1186/s11689-022-09442-0 PMC9128253

[43] Hurst JH , Liu Y, Maxson PJ, et al Development of an electronic health records datamart to support clinical and population health research. J Clin Transl Sci. 2020;5:e13. 33948239 10.1017/cts.2020.499 PMC8057430

[44] Epic Systems Corporation. Epic. Epic Systems Corporation; 2023.

[45] Stolte A , Merli MG, Hurst JH, et al Using electronic health records to understand the population of local children captured in a large health system in Durham county, NC, USA, and implications for population health research. Soc Sci Med. 2022;296:114759. 35180593 10.1016/j.socscimed.2022.114759 PMC9004253

[46] Alsentzer E , Murphy JR, Boag W, et al Publicly available clinical BERT embeddings [Internet]. arXiv, 2019 [cited 2025 May 18], http://arxiv.org/abs/1904.03323, preprint: not peer reviewed.

[47] Fatima S , Ali S, Kim HC. A comprehensive review on multiple instance learning. Electronics (Basel). 2023;12:4323.

[48] Lundberg S , Lee SI. A unified approach to interpreting model predictions Advances in Neural Information Processing Systems, 2017: 4765--4774

[49] Owen G. Values of games with a priori unions. In: R Henn, O Moeschlin, eds. Essays in Mathematical Economics and Game Theory. Springer; 1977:76-88.

[50] Python Software Foundation. Python [Internet]. Python Software Foundation. 2024 [cited 2025 May 18]. https://www.python.org/downloads/release/python-3119/

[51] R Core Team. R: a language and environment for statistical computing [Internet]. R Foundation for Statistical Computing. 2022 [cited 2025 May 18]. https://www.R-project.org/

[52] World Health Organization. International Classification of Diseases, Tenth Revision (ICD-10). 10th ed. World Health Organization. 2019.

[53] Scherr CL , Getachew-Smith H, Moe S, et al Possible unintended consequences of pediatric clinician strategies for communicating about social-emotional and developmental concerns in diverse young children. Fam Syst Health. 2024;42:18-33. 38647491 10.1037/fsh0000882 PMC11070196

[54] Lindgren Fändriks A , Almqvist K, Hjärthag F, et al Child health care nurses’ experience of language screening for 2.5‐year‐old children: a qualitative study. Nurs Open. 2023;10:6583-6591. 37317482 10.1002/nop2.1918 PMC10416044

[55] Zubler JM , Wiggins LD, Macias MM, et al Evidence-Informed milestones for developmental surveillance tools. Pediatrics. 2022;149:e2021052138. 35132439 10.1542/peds.2021-052138 PMC9680195

[56] McLaughlin MR. Speech and language delay in children. Am Fam Physician. 2011;83:1183-1188. 21568252

[57] Nelson BB , Ratushnyak D, Richards A, et al Using claims data to map unmet service needs for early childhood developmental disabilities in Virginia. Acad Pediatr. 2023;23:457-463. 36108999 10.1016/j.acap.2022.09.003 PMC10008751

[58] Chua WD , Pipan M, Rosen AR. Neurologic and developmental considerations in children with medical complexity. Curr Probl Pediatr Adolesc Health Care. 2021;51:101073. 34657814 10.1016/j.cppeds.2021.101073

[59] Leyenaar JK , Arakelyan M, Schaefer AP, et al Neurodevelopmental and mental health conditions in children with medical complexity. Pediatrics. 2024;154:e2024065650. 39099441 10.1542/peds.2024-065650 PMC11350095

