# Supplementary material for: Using clinical notes to identify children with speech-language delay and understand differences in diagnostic timing
Source: JAMIA Open. 2026 Jun 12;9(3):ooag090. doi: 10.1093/jamiaopen/ooag090 (PMC13264438; doi:10.1093/jamiaopen/ooag090)
Supplement: ooag090_Supplementary_Data [file ooag090_supplementary_data.docx]

| **Supplemental Table 1.** ICD-10 Codes Used for Cohort Selection and Analysis | | |
| --- | --- | --- |
| **Code Type** | **ICD-10 Codes** | **Descriptor** |
| Speech-language Delay Diagnosis | F80.0 | Phonological disorder |
|  | F80.1 | Expressive language disorder |
|  | F80.2 | Mixed receptive-expressive language disorder |
|  | F80.4 | Speech and language development delay due to hearing loss |
|  | F80.8 | Other developmental disorders of speech and language |
|  | F80.81 | Childhood onset fluency disorder |
|  | F80.89 | Other developmental disorders of speech and language |
|  | F80.9 | Developmental disorder of speech and language, unspecified |
|  | R47.82 | Fluency disorder in conditions classified elsewhere |
|  | R47.89 | Other speech disturbances |
|  | R47.9 | Unspecified speech disturbances |
|  | R62.0 | Delayed milestone in childhood |
|  | H93.25 | Central auditory processing disorder |
| Well-Child Visit | Z00.110 | Health examination for newborn under 8 days old |
|  | Z00.111 | Health examination for newborn 8 to 28 days old |
|  | Z00.121 | Encounter for routine child health examination with abnormal findings |
|  | Z00.129 | Encounter for routine child health examination without abnormal findings |
| Motor Delay | 315.4 (ICD-9) | Developmental coordination disorder |
|  | 781.2 (ICD-9) | Abnormality of gait |
|  | 781.3 (ICD-9) | Lack of coordination |
|  | 783.42 (ICD-9) | Delayed milestones |
|  | F82 | Specific developmental disorder of motor function |
|  | R26.9 | Unspecified abnormalities of gait and mobility |
|  | R27.9 | Unspecified lack of coordination |
|  | R29.898 | Other symptoms and signs involving the musculoskeletal system |
|  | R48.2 | Apraxia |
|  | R62.0 | Delayed milestone in childhood |
| Developmental Delay | 315.5 (ICD-9) | Mixed development disorder |
|  | 315.8 (ICD-9) | Other specified delays in development |
|  | 783.4 (ICD-9) | Lack of expected normal physiological development in childhood |
|  | 783.42 (ICD-9) | Delayed milestones |
|  | F81.9 | Developmental disorder of scholastic skills, unspecified |
|  | F88 | Code for Other disorders of psychological development |
|  | R62.0 | Delayed milestone in childhood |
|  | R62.50 | Unspecified lack of expected normal physiological development in childhood |
| Autism Spectrum Disorder | 299 (ICD-9) | Pervasive developmental disorders |
|  | 299.8 (ICD-9) | Other specified pervasive developmental disorders |
|  | F84.0 | Autistic disorder |
|  | F84.5 | Asperger's syndrome |
|  | F84.8 | Other pervasive developmental disorders |
|  | F84.9 | Pervasive developmental disorder, unspecified |

| **Supplemental Table 2.** Hyperparameter Options Evaluated During Model Tuning | |
| --- | --- |
| **Hyperparameters** | **Choices** |
| Epoch | 3, 4, 5, 6, 7, 8 |
| Chunk size | 64, 128, 256 |
| Chunk overlap size | 32, 64, 128 |
| Dropout rate | 0.001, 0.01, 0.1 |
| Learning rate | 1e-5, 3e-5, 5e-5, 1e-4 |

| **Supplemental Table 3.** Baseline Characteristics of Speech-language Delay and Matched Control Children in the NLP Model Learning Cohort | | | | |
| --- | --- | --- | --- | --- |
| **Variable** | **Level** | **Case (Speech-language Delay)** | **Control (No speech-language Delay)** | **SMD** |
| *N* |  | 1733 | 1733 |  |
| Age [Q1, Q3] |  | 20.1 [18.1, 25.0] | 20.0 [18.1, 25.0] | 0.085 |
| WC visits (median (IQR))^a^ |  | 8.0 [7.0, 9.0] | 8.0 [7.0, 9.0] | 0.017 |
| 12-month OP visits (median (IQR))^b^ |  | 6.0 [4.0, 10.0] | 6.0 [4.0, 10.0] | 0.030 |
| SEX (%) | Female | 594 (34.3) | 594 (34.3) | <0.001 |
|  | Male | 1139 (65.7) | 1139 (65.7) |  |
| RACE_ETH (%) | NH-White | 481 (27.8) | 481 (27.8) | <0.001 |
|  | NH-Black | 581 (33.5) | 581 (33.5) |  |
|  | NH-Asian | 60 (3.5) | 60 (3.5) |  |
|  | Hispanic | 456 (26.3) | 456 (26.3) |  |
|  | Others | 155 (8.9) | 155 (8.9) |  |
| Language (%) | English | 1410 (81.4) | 1435 (82.8) | 0.049 |
|  | Spanish | 307 (17.7) | 278 (16.0) |  |
|  | Other | 16 (0.9) | 20 (1.2) |  |
| Payer (%) | Private | 644 (37.2) | 644 (37.2) | <0.001 |
|  | Public | 1050 (60.6) | 1050 (60.6) |  |
|  | Self-Pay or Special | 39 (2.3) | 39 (2.3) |  |
| Co-Occurring Conditions (%) | MD | 145 (8.4) | 75 (4.3) | 0.166 |
|  | DD | 64 (3.7) | 25 (1.4) | 0.143 |
|  | ASD | 0 (0) | 1 (0.1) | 0.034 |
| ADI (%)^c^ | 1 | 191 (11.0) | 183 (10.6) | 0.095 |
|  | 2 | 328 (19.0) | 323 (18.6) |  |
|  | 3 | 231 (13.4) | 223 (12.9) |  |
|  | 4 | 228 (13.0) | 209 (12.1) |  |
|  | 5 | 198 (11.4) | 228 (13.2) |  |
|  | 6 | 90 (5.2) | 88 (5.1) |  |
|  | 7 | 109 (6.3) | 106 (6.1) |  |
|  | 8 | 92 (5.3) | 94 (5.4) |  |
|  | 9 | 112 (6.5) | 95 (5.5) |  |
|  | 10 | 67 (3.8) | 74 (4.3) |  |
|  | Unknown | 87 (5.0) | 110 (6.3) |  |
| Abbreviations: SMD, Standardized Mean Difference; WC, Well-Child; OP, outpatient; ETH, Ethnicity; NH, Non-Hispanic; MD, Motor Delay; DD, Developmental Delay; ASD, Autistic Spectrum Disorders; ADI, Area Deprivation Index.  ^a^Number of all previous Well-Child visits.  ^b^Number of outpatient visits in previous 12 months.  ^c^Lower ADI represents wealth or prosperity, while higher ADI reflects deprivation. | | | | |


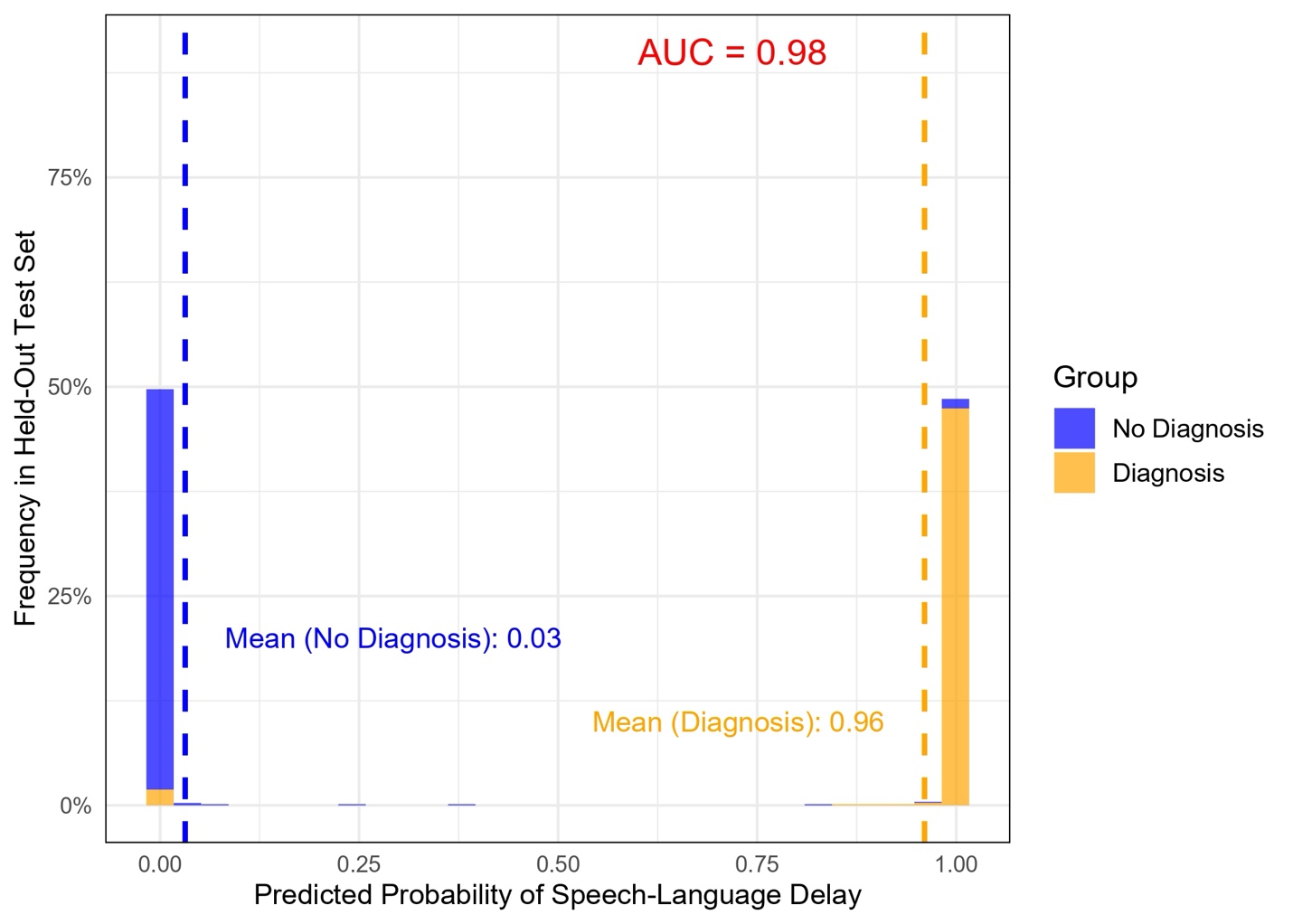


**Supplemental Figure 1.** Distribution of predicted probabilities for speech-language delay by Model on Held-out Test Set of Learning Cohort. Blue bars represent where there is no associated ICD-10 code, while yellow bars represent where there is an associated code. Overall, the generated model is very confident (most predicted probabilities are close to 0 or 1) and accurate. However, there is a small subset of encounters where there is evidence of speech-language delay documentation but no associated code.
